# Effect of over-the-counter brimonidine tartrate 0.025% ophthalmic solution on pupil size in healthy adults

**DOI:** 10.1007/s00417-021-05297-8

**Published:** 2021-07-12

**Authors:** Mitra Nejad, Shawn R. Lin, Linda H. Hwang, Mark Landig, Saba Al-Hashimi, John D. Bartlett

**Affiliations:** Stein Eye Institute, David Geffen School of Medicine, University of CA – Los Angeles, 300 Stein Plaza, Los Angeles, CA 90095 USA

**Keywords:** Brimondine tartrate 0.025%, Pupillary miosis, Dysphotopsia

## Abstract

**Purpose:**

To evaluate the effect of brimonidine tartrate 0.025% ophthalmic solution on pupil size under scotopic conditions in healthy adults

**Methods:**

Pupil size was measured in 56 eyes of 28 volunteer participants using a pupillometer under scotopic conditions. Age, gender, and iris color were recorded. Subjects using any ophthalmic medications other than artificial tears were excluded. The pupil size was subsequently measured again under scotopic conditions 60 min after instillation of brimonidine tartrate 0.025% ophthalmic solution.

**Results:**

Statistically significant miosis was seen after instillation of brimonidine tartrate 0.025% (p = 0.04). Average pupil size prior to brimonidine 0.025% instillation was 7.28 ± 1.05 mm, and average pupil size after instillation of brimonidine 0.025% was 6.36 ± 1.68 mm, a reduction of − 23.7% in pupil area. Subjects with light irides demonstrated a greater miotic effect than subjects with dark irides (1.55 mm vs. 0.67 mm, p < 0.0001), with a pupil area reduction of − 37.6% and − 17.4%, respectively. The amount of miosis was independent of initial pupil size.

**Conclusions:**

Brimonidine tartrate 0.025% causes significant miosis in scotopic settings, although the effect is not as great in darker colored eyes. Further studies are needed to determine the latency and duration of the effect and whether the amount of miosis is clinically significant.


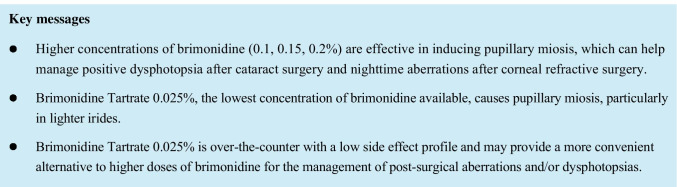


## Introduction

Brimonidine tartrate 0.025% ophthalmic solution, a selective alpha-2 adrenergic receptor agonist, is FDA-approved for relieving redness of the eye. It has been available for over-the-counter use in the USA as Lumify® (Bausch & Lomb) since 2018. Higher doses of brimonidine (0.1%, 0.15%, and 0.2%) have been available by prescription since 1996, primarily for the treatment of glaucoma or ocular hypertension. Several studies have shown that these higher doses of brimonidine can cause pupillary miosis [1–8].

Laser in situ keratomileusis can induce spherical aberration and nighttime halos due to discrepancies between the optical zone and scotopic pupil size, and these aberrations and distortions have been effectively treated with pharmacologic miosis [9, 10]. Pupillary constriction has also been shown to be an effective treatment for positive dysphotopsias after cataract surgery. These undesired light streaks, starburst, arcs, and flashes are caused by light striking the edge of the intraocular lens implant and may potentially be dampened by decreasing the size of the pupil [11].

Higher doses of brimonidine (both 0.15 and 0.2%) have been effectively utilized for treatments of some of these post-surgical symptoms [10–12]. However, these eye drop must be prescribed and are associated with a number of side effects, most commonly allergic conjunctivitis [13, 14]. In contrast, participants in phase III studies on brimonidine tartrate 0.025% rated the drop as “very comfortable” [15]. To our knowledge, there has not been a study measuring the effect of brimonidine tartrate 0.025% on pupil size, a potentially convenient and low side effect profile alternative to higher concentration brimonidine therapy.

## Methods

Institutional Review Board (IRB)/Ethics Committee approval was obtained at the University of California—Los Angeles prior to the initiation of this study (IRB #19–001,190). Informed consent was obtained for every study participant. Persons who were pregnant, breastfeeding, or actively using any ocular medications were excluded. Age and self-reported iris color were recorded. Pupil size was measured using a Keeler PupilScan II Pupillometer (Keeler, Malvarn, PA) in a scotopic setting. Volunteers were asked to look 20 ft away to avoid accommodation during measurements. One drop of brimonidine tartrate 0.025% was instilled in both eyes by the examiner. All participants waited in a dimly lit auditorium while listening to lectures. Pupil size was measured again after 60 min. All measurements were done in the same dark room for all participants, and lighting conditions were kept unchanged after the 60-min interval. Patients were asked to report any adverse symptoms they may have experienced during that interval. After collecting data on 11 participants (22 eyes), statistical analysis was performed using a Student T-test to compare groups. It was determined that a minimum of 50 eyes was needed to achieve sufficient statistical power. Statistical analysis and p values were calculated using Microsoft Excel.

## Results

A total of 56 eyes of 28 volunteers were tested. Of these, 40 eyes were reported as brown, 10 as blue, and 6 as either hazel or green. Average age was 39.7 years ± 1.77 years with range between 22 and 84 years old. There were 12 females and 16 males included in the study. Descriptive statistics can be found in Table 1.

**Table 1:** Descriptive statistics

Parameter	Number
No. of eyes	56
No. of patients	28
Age (mean ± SD) (year)	39.7 ± 1.8
Sex (eyes)	
Male	24
Female	32
Iris color (eyes)	
All	56
Brown	40
Blue	10
Green/hazel	6

**Fig. 1:**
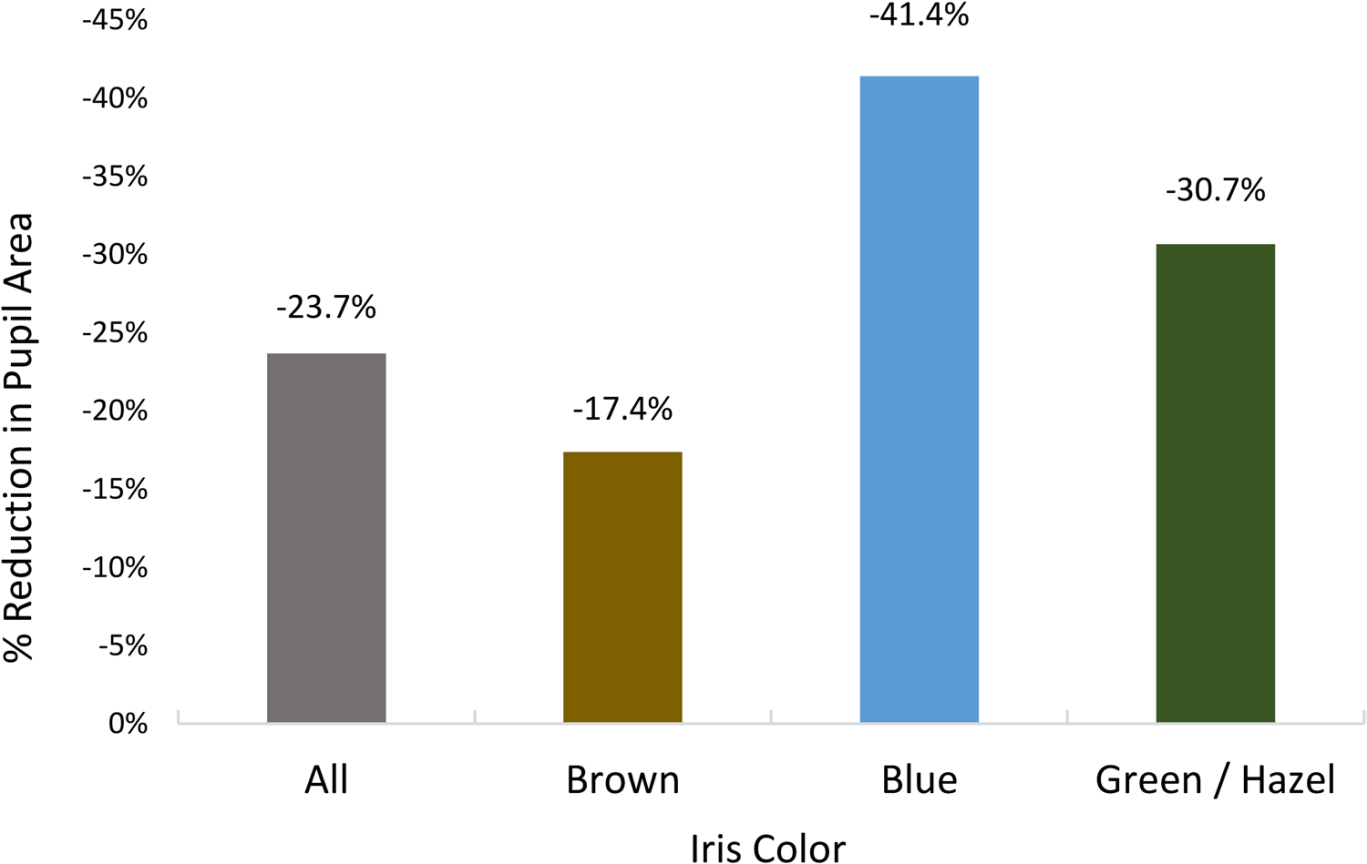
The percentage reduction in pupil area by iris color

Pupil size results are reported in Table 2. The overall pupil size after instillation of brimonidine 0.025% decreased significantly from 7.28 to 6.36 mm, a difference in pupil area of − 23.7% (p < 0.0001). Figure 1 demonstrates the percent reduction of pupil size by iris color. Lighter colored iris subgroups also showed significant reductions in pupil area, with blue irides showing a 41.4% reduction in pupil area (p = 0.001) and green/hazel irides showing a 30.7% reduction (p = 0.08).

**Table 2:** Pupillary miosis with brimonidine 0.025% instillation

	Total	Pre-instillation (mm)	Post-instillation (mm)	Difference (mm)	Reduction in pupil area (%)	*P* value
Iris color (eyes)						
All	56	7.28	6.36	− 0.91	− 23.7%	p < 0.0001
Brown	40	7.24	6.58	− 0.67	− 17.4%	p = 0.005
Blue	10	7.46	5.71	− 1.75	− 41.4%	p = 0.001
Green/hazel	6	7.23	6.02	− 1.22	− 30.7%	p = 0.08
Iris color groups						
Dark (brown)	40	7.24	6.58	− 0.67	− 17.4%	Dark vs. light p < 0.0001
Light (blue, green, hazel)	16	7.38	5.83	− 1.55	− 37.6%	
Pupil size groups						
Top 50%	26	7.99	6.95	− 1.04	− 24.3%	Top vs. bottom p = 0.11
Bottom 50%	26	6.56	5.77	− 0.79	− 22.6%	
Top 25%	26	8.29	7.1	− 1.19	− 26.6%	Top vs. bottom p = 0.17
Bottom 25%	26	6.00	5.10	− 0.9	− 27.8%

**Fig. 2:**
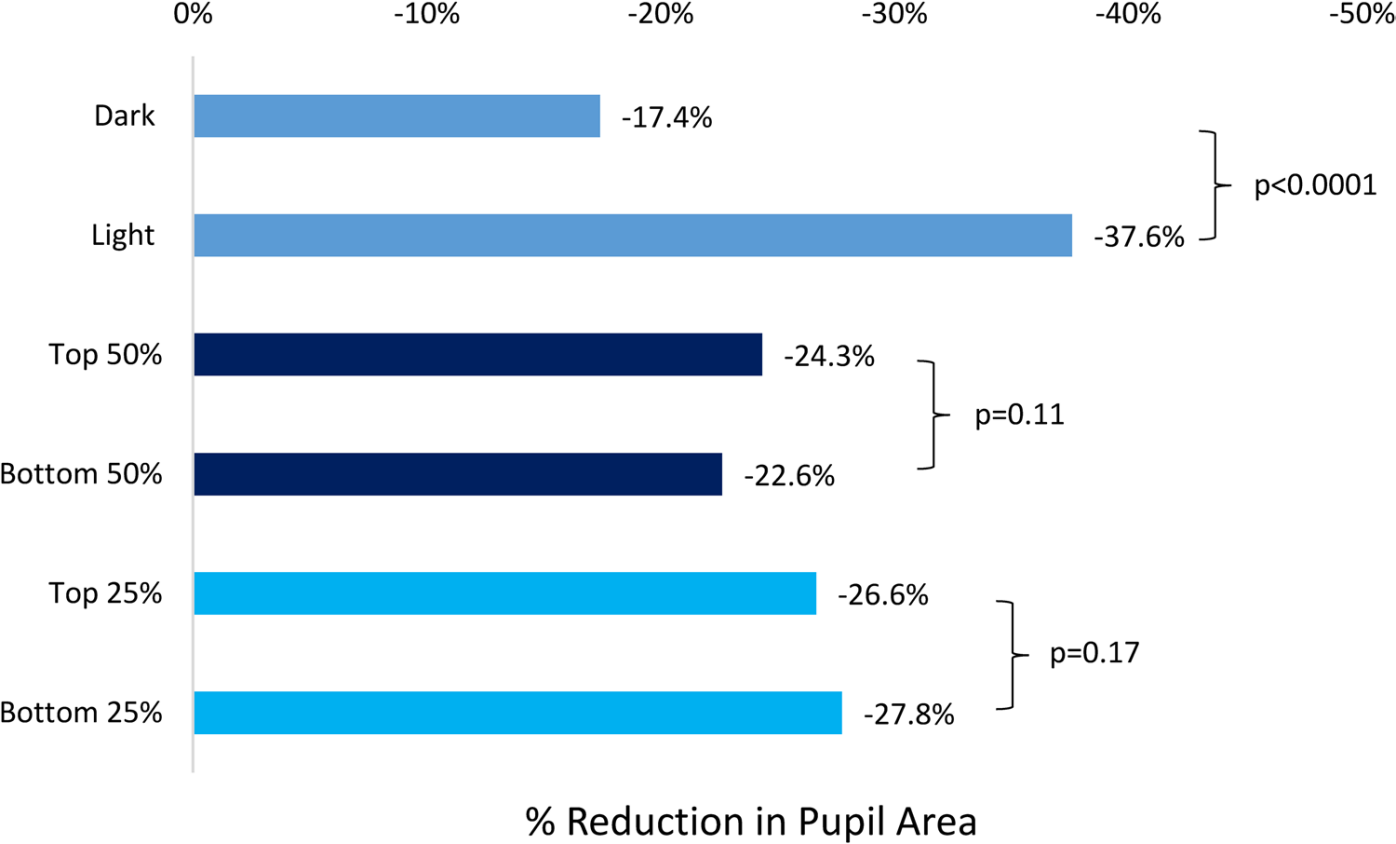
The percentage reduction of pupil area for dark vs. light irides, the top 50% of initial pupil size vs. bottom 50% of initial pupil size, and the top 25% of initial pupil size vs. bottom 25% of initial pupil size

Several grouped analyses were performed comparing lighter colored irides (blue, green, and hazel) with darker (brown) irides and showed significantly greater miosis in the lighter colored group (p < 0.0001, Fig. 2). Pupillary area reduction of the largest (top 50% and top 25%), pupil pre-instillation with the smallest (bottom 50% and 25%), and pupil pre-instillation (p = 0.11, and p = 0.17, respectively) were compared. There was not a significant difference in post-instillation pupillary area reduction between the two groups. Finally, to assess the effect of age on the amount of pupillary miosis, the oldest half of study participants was compared to the youngest half of study participants. Again, there was no statistically significant difference in post-instillation pupillary area reduction between the two age groups. To help further confirm these observed trends, the data was reanalyzed using one eye per participant selected randomly. A statistically significant reduction in pupil size was seen when analyzed for all 28 eyes as well as for subgroups of brown and blue eyes (Table 3).

**Table 3:** Pupillary miosis with brimonidine 0.025% instillation (random eye chosen)

	Total	Pre-instillation (mm)	Post-instillation (mm)	Difference (mm)	Reduction in pupil area (%)	*P* value
Iris color (eyes)						
All	28	7.31	6.37	− 0.94	− 24.1%	p < 0.002
Brown	20	7.28	6.63	− 0.66	− 17.2%	p = 0.04
Blue	5	7.44	5.54	− 1.9	− 44.6%	p = 0.01
Green/hazel	3	7.27	6.03	− 1.24	− 31.2%	p = 0.19

There were no adverse events reported.

## Discussion

Phase III efficacy studies found brimonidine 0.025% (Lumify) to be safe and effective for reduction of ocular redness, with an 8-h duration of action, no evidence of tachyphylaxis, and negligible rebound redness [15]. Overall, it was well-tolerated with minimal adverse events reported. In one clinical trial, only 4 patients of 40 using brimonidine tartrate 0.025% reported adverse symptoms (pruritus, foreign body sensation, increased lacrimation, and pain—further characterized as mild stinging). The eye drop was rated as very comfortable by patients and found to be safe with only one reported treatment-related ocular adverse event (mild pain) which resolved spontaneously despite continued use of the drop.

Of note, in the brimonidine tartrate 0.025% (Lumify) clinical trials, lowering of intraocular pressure was not observed, raising the question of whether other effects of higher dose brimonidine, such as pupil constriction and/or eyelid elevation, would also be seen with brimonidine tartrate 0.025%. At the time of this study, phase IV clinical trials on the effect of brimonidine tartrate 0.025% on eyelid position are underway. To our knowledge, this is the first study on the effect of brimonidine tartrate 0.025% on pupil size. Our study demonstrated a statistically significant pupillary miosis 60 min after instillation of the drop.

**Fig. 3:**
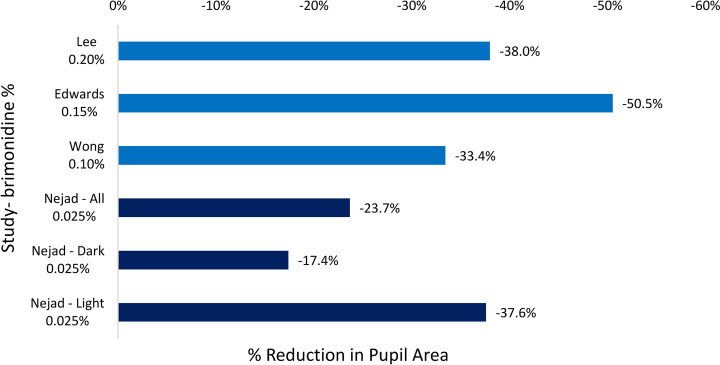
Comparison of percent reduction in pupil area of three other published studies compared to current study (all irides, dark irides, and light iridis)

Pupil constriction has been shown to be an effective strategy for management of both halos after laser vision correction and positive dysphotopsias after cataract surgery. A number of prescription ophthalmic solutions have demonstrated pharmacologic miosis, including parasympathomimetic drops—such as pilocarpine—as well as three different concentrations of brimonidine. The effectiveness of pilocarpine in inducing pupillary miosis is well-established—one study demonstrated 3.23 mm (− 83.1%) reduction after 90 min with 2% pilocarpine [16]. However, its clinical use has been limited due to its side effect profile, which could include eye irritation; headache; dyspnea; nausea; excessive sweating or salivation; and, rarely, retinal detachment [17, 18]. In one study using brimonidine 0.2% on post-LASIK patients with night vision symptoms, Lee et al. found a 1.36-mm pupillary constriction after 1 h. They used a computerized model to quantify halo size in these patients and found a statistically significant (29%) decrease in halo size after use of brimonidine 0.2% [10]. Edwards et al. conducted a study on brimonidine 0.15% and post-laser vision correction patients. They found pupillary miosis from 6.44 ± 1.11 mm to 4.53 ± 1.27 mm after 1 h (− 38.0%) [12]. This change was associated with improved contrast sensitivity and a subjective decrease in night driving difficulty. Finally, brimonidine 0.1% (the lowest available strength of brimonidine prior to Lumify) showed a mean constriction of 1.35 mm (− 33.4%) in one study [8]. This was associated with a statistically significant decrease in RMS higher order aberrations, suggesting that brimonidine 0.1% may be effective in treating symptoms caused by these aberrations. These studies are summarized in Fig. 3.

While these studies show the clinical efficacy of brimonidine, there is not a specific amount of minimum pupillary miosis known to be necessary to dampen aberrations. It is unclear whether the 0.9-mm average pupillary miosis seen in our study would be clinically significant; however, in light eyes, we found 1.55 mm of constriction (− 37.6%), on par with what was seen in the studies reviewed above with both 0.1% and 0.2% concentrations of brimonidine. This is suggestive that for light iridis, brimonidine tartrate 0.025% may cause enough pupillary miosis to reduce halo size and higher order aberrations, and to improve contrast sensitivity and night driving ability as seen in these studies. While a higher concentration of brimonidine is available for symptomatic post-surgical patients, these formulations require a prescription and can often be intolerable due to ocular allergies. Brimonidine tartrate 0.025% may provide a convenient alternative that is available over-the-counter with a better side effect profile than higher dose brimonidine.

While our study found approximately 1 mm of pupil constriction with brimonidine tartrate 0.025%, further studies will be needed to assess whether the amount of pupillary miosis seen would result in reduction in halo size, higher order aberrations, and contrast sensitivity and/or would be associated with a reduction of subjective symptoms of halos or positive dysphotopsias. There are a number of other limitations in our study. The first is the relatively small sample size. A larger sample would be needed to further confirm the trends we saw regarding age and iris color. The miotic effect seen may increase with a larger sample size. A second limitation is that we used both eyes from each individual participant, potentially doubling our statistical power. This was addressed by reanalyzing the data with a randomly selected eye from each participant, which revealed the same statistically significant trends. A randomized placebo-controlled or a contralateral eye-controlled trial would be optimal for confirming the veracity of the results. While many studies have shown brimonidine to affect pupil size within 1 h and last for up to 6 h, checking pupil size at later intervals would be needed to assess any continued progression of pupillary constriction beyond 1 h as well as to measure total constriction duration. In brimonidine tartrate 0.025% clinical trials, the reduction of ocular redness was shown to last up to 8 h. Further studies would be needed to assess the frequency of instillation needed to maintain pupillary miosis.

In conclusion, our study demonstrated statistically significant miosis 1 h after instillation of brimonidine tartrate 0.025% in scotopic conditions with a trend towards more miosis in blue eyes. Brimonidine tartrate 0.025% is available over-the-counter as Lumify®. It may be a more convenient alternative to higher dose brimonidine or pilocarpine for the purpose of pupillary constriction to achieve reduction of halos, dysphotopsias, or night vision problems in symptomatic patients who have had refractive or cataract surgery.

## Data Availability

All data collected can be made available for review upon request.
